# Child Exposure to Lead in the Vicinities of Informal Used Lead-Acid Battery Recycling Operations in Nairobi Slums, Kenya

**DOI:** 10.5696/2156-9614-6.12.15

**Published:** 2016-12-22

**Authors:** Maureene Auma Ondayo, Gelas Muse Simiyu, Phillip Okoth Raburu, Faridah Hussein Were

**Affiliations:** 1 Department of Environmental Health and Biology, School of Environmental Studies, University of Eldoret, Eldoret, Kenya; 2 Department of Fisheries and Aquatic Sciences, School of Natural Resource Management, University of Eldoret, Eldoret, Kenya; 3 Department of Chemistry, University of Nairobi, Nairobi, Kenya

**Keywords:** soil, house dust, predicted child blood lead, used lead-acid battery recycling, Nairobi slums, IEUBK

## Abstract

**Background.:**

Child exposure to lead from informal used lead-acid battery (ULAB) recycling operations is a serious environmental health problem, particularly in developing countries.

**Objectives.:**

We investigated child exposure to lead in the vicinities of ULAB recycling operations in the Dandora, Kariobangi and Mukuru slums in Nairobi between January and August 2015.

**Methods.:**

Top soil (n = 232) and floor dust (n = 322) samples were collected from dwelling units (n = 120) and preparatory schools (n = 44) and analyzed using an inductively coupled plasma-optical emission spectrometer at the Mines and Geological Department Laboratory in the Ministry of Mining, Nairobi. From the obtained lead levels in soil and house dust, child blood lead levels were subsequently predicted using the Integrated Exposure Uptake Biokinetic Model for Lead in Children (IEUBK), Windows version.

**Results.:**

Lead loadings in all the floor dust samples from the Dandora, Kariobangi and Mukuru slums exceeded the United States Environmental Protection Agency (USEPA) guidance value for lead on floors with a range of 65.2 – 58,194 μg/ft^2^. Control floor dust samples recorded lower lead loadings compared to the Dandora, Kariobangi and Mukuru slums. Lead concentration in 70.7% of the soil samples collected from waste dumps, industrial sites, residential areas, playgrounds and preparatory schools in Dandora, Kariobangi and Mukuru exceeded the respective USEPA guidance values for lead in soils. Lead concentration in 100% of control soil samples were below the respective USEPA limits. The IEUBK model predicted that nearly 99.9% of children ≤ 7 years old living near informal ULAB recycling operations in Dandora, Kariobangi and Mukuru were at risk of being lead poisoned, with predicted blood lead levels (BLL) above the Centers for Disease Control (CDC) reference value for blood lead. A total of 99.9% of exposed children living in the Mukuru slums are likely to have BLL above 34 μg/dL.

**Conclusions.:**

There is a need for coordinated efforts to decrease lead emissions from informal battery recycling in Nairobi slums and to remediate existing soils, particularly around battery workplaces and dumpsites. The BLL of local children should be clinically tested and appropriate intervention measures taken.

## Introduction

Lead exposure from informal used lead-acid battery (ULAB) recycling operations is a serious environmental health problem, especially in developing countries.^1–3^ Research shows that young children living near informal ULAB recycling operations have elevated blood lead levels (BLL) and fatalities have also been reported.^4–7^ The operations release large amounts of lead dust and wastes containing lead into surrounding soils, air, buildings and waterways.^1^,^4^,^6^,^8^ The released lead ultimately finds its way into human bodies through various pathways.^2^,^5^,^6^ In young children, ingestion of lead-contaminated soil and dust is the major pathway of exposure. Young children are usually the most exposed and susceptible to the toxic effects of lead due to their behavioral, physiological and developmental characteristics.^9–11^ Lead is highly toxic and once in the child's body, it severely and perhaps permanently interferes with growth, differentiation and developmental processes of vital organs and systems such as the brain, blood tissue, kidneys and the skeleton, and may lead to death.^3^,^12–14^

Nairobi is the capital city of Kenya with a population of about 4 million and a gross national income of US $1,280 per capita.^15^ Several unregulated informal ULAB recycling operations are carried out in the city, particularly in the densely populated slum areas, including slums in Kibera, Dandora, Kariobangi and Mukuru. Previous studies in Kenya assessed occupational exposure to lead in the formal battery recycling sectors and found significant levels of lead in human (blood, hair, nails) and environmental (soil, air and water) samples.^16^,^17^ To date, no studies have been carried out in the informal battery recycling sector in Nairobi. The present study therefore assessed child exposure to lead in the vicinities of informal ULAB recycling operations in Dandora, Kariobangi and Mukuru slums in Nairobi in an effort to characterize childhood lead exposure in these communities. The study focused on young children between 0–7 years (0–84 months), and had the following specific objectives:

**1.** To determine the levels of lead in floor dust in dwelling units and preparatory schools in the vicinities of informal ULAB recycling operations in Nairobi slums.
**2.** To determine the concentration of lead in soil in residential areas, preparatory schools, children's playgrounds and dumpsites in the vicinities of informal ULAB recycling activities and workplaces.
**3.** To predict BLL in children under seven years of age living within the study area using the Integrated Exposure Uptake Biokinetic Model for Lead in Children (IEUBK), Windows version.^18^,^19^

The overall goal was to gather information that could be used by policy makers and relevant stakeholders to protect children from the risks associated with ULAB recycling in Nairobi.

**Abbreviations**
*BLL*: Blood lead level
*IEUBK*: Integrated Exposure Uptake Biokinetic Model for Lead in Children
*ULAB*: Used lead-acid battery
*USEPA*: United States Environmental Protection Agency

## Methods

**Figure 1:**
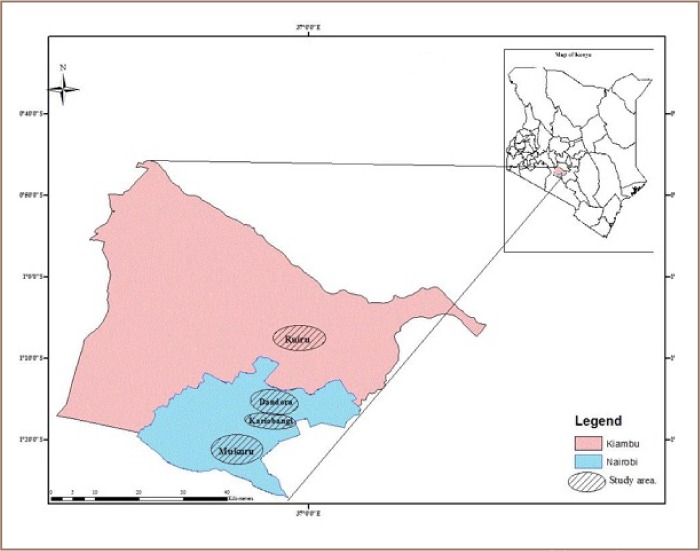
Map of the Study Area

This cross sectional study was carried out from January to August, 2015. The study area was purposively selected based on the presence of informal ULAB recycling activities that were suspected to be potential sources of childhood exposure to lead. A control area with similar traffic and industrial activity was chosen in Ruiru as shown in Figure 1.

In this study, a dwelling unit refers to a non-conventional, slum-type low-cost housing unit usually constructed with non-conventional materials which are obtained through informal means. On average, each dwelling unit had seven occupants. Dwelling units were deemed eligible if children between 0 to 84 months of age resided there. Dwelling units and preparatory schools were excluded if they were painted to avoid the confounding effect of lead in paint. Indoor dust referred to dust settled on floors inside dwelling units and preparatory schools. Random, yet representative, indoor dust sampling points were selected as follows: dwelling units with children below seven years of age and preparatory schools where children spent most of their time learning or under care. During the study period, there were no informal ULAB recycling operations or other known point sources of lead in the control community (Ruiru).

### Sample Collection and Preparation

#### Floor Dust

The US Department of Housing and Urban Development dust wipe method was followed during collection and preparation of floor dust samples.^20^ The method determines lead loadings on surfaces, allows for comparisons, and samples the surface that most likely is the source of children's exposure to lead laden dust. Results from dust wipe analyses correlate with children's BLL and can therefore be suitably used in the model to make predictions.^20^,^21^ The midpoint or largest area in the room was selected for floor dust sampling unless the children had a specific play area in the room, in this case the play area was considered. A one square foot plastic template was carefully placed on the sampling area on the floor without disturbing the dust. The outside edges of the template were taped to the floor to keep it from moving while wiping. Using preweighed moist wipes (Cussons baby wipes, UK), dust samples were collected from the surface covered by the template. The samples were transferred into clean preweighed Ziploc polyethene bags, and then labeled. A total of 254 floor dust samples were collected from dwelling units and preparatory schools in the Dandora, Kariobangi and Mukuru slums. Using the same standard procedures, 78 floor dust samples were collected from dwelling units and preparatory schools in the control community (Ruiru). All of the 322 indoor floor dust samples were taken to the Mines and Geological Department Laboratory in the Ministry of Mining in Nairobi. Each dust sample was weighed together with the wipes and Ziploc polyethene bags. For each sample, total weight of dust collected (dust loading) was determined by the difference between the weight of the wipe and the polyethene bag, and recorded.

#### Soil

Soil sampling points were randomly selected in children's environments defined as 1) industrial sites where informal ULAB recycling was carried out; 2) waste dumps where waste from the informal ULAB recyclers was disposed; 3) residential areas where young children resided; 4) playgrounds where young children spent most of their time playing; and 5) preparatory schools where infants and children spent most of their time while under care or/and learning.

Using a clean plastic shovel, 232 top soil samples were collected 0–2.5 cm deep from soil in residential areas, preparatory schools, playgrounds, industrial area and waste dumps. In each case, three individual soil samples were collected from different spots then combined to form one sample. The plastic shovel was decontaminated before each subsequent sampling. Each soil sample was kept in a separate clean Ziploc polyethene bag, labeled, and taken to the Mines and Geological Department Laboratory in the Ministry of Mining. The soil sample was sieved, then oven-dried at 40°C to a constant weight, crushed, and homogenized prior to chemical digestion.

### Sample Digestion

#### House Dust

After homogenizing each house dust sample, a 2.5 g representative was weighed using an electronic analytical balance (Kern & Sohn, GMbH, Germany) and placed into a 50 mL glass beaker then 20 mL de-ionized water added and the sample placed in the fume extraction hood. Next, 20 mL of 69% concentrated nitric acid was added. After 8 hours, 2 mL of 37% concentrated hydrochloric acid and 3 mL of 30% hydrogen peroxide were added and the contents allowed to react for approximately 5 minutes, then heated on a hot plate to 180°C–200°C for 5 minutes. The temperature was maintained for 10 minutes, then the beaker contents allowed to cool. On cooling, the beaker contents were filtered, diluted with de-ionized water and brought up to volume in a 50 mL volumetric flask prior to analysis.^22^ This is a typical digestion process to release lead from the dust wipe matrix and the soil matrix.

### Soil Sample

For each soil sample, 0.5 g was weighed and placed into an inert polymeric microwave digestion vessel (Multiwave 3000, Anton Paar GmbH, Germany). The vessels were placed in the fume hood, then 5.0 mL of double distilled water, 9.0 mL of concentrated nitric acid (65%), 1.0 mL of concentrated hydrochloric acid (30%), and 2.0 mL hydrogen peroxide (30%) were added. Double distilled water was added to improve mineral solubility and prevent temperature spikes due to exothermic reactions. In order to allow gases to escape, each sample was allowed to react for approximately 5 minutes prior to sealing. The vessels were then placed on the rotor and placed in the microwave and heated between 180°C and 210°C for 5.5 minutes, then maintained at the same temperature for another 15 minutes. After cooling, the vessel contents were filtered and diluted with double distilled water to a volume of 100 mL in a volumetric flask prior to analysis.

### Analysis of Lead

Standard working solutions were prepared from commercial stock solutions in order to calibrate the inductively coupled plasma-optical emission spectrometer (ICP OES). Calibration curves were produced with a standards concentration range of 0.0 mg/kg - 20.0 mg/kg. Lead concentrations in soil and floor dust digests were determined using the calibrated ICP OES (Spectro Arcos ICP Model FHS12, Germany). The method detection limit for lead was calculated as three times the standard deviation for the digestion blanks (n=5).^23^

### Quality Control and Assurance

Detailed standard procedures for collection, transport, and storage of samples were followed.^20^ Analytical grade chemicals (Sigma-Aldrich Co, Germany) were used throughout the analyses. Deionized water was used throughout the analytical procedures.

Floor dust field blanks were prepared following similar procedures for collection of floor dust.^20^ Lead concentrations in the floor dust field blanks ranged from not detected to 2.1 μg/ft^2^. Laboratory wipe sample blanks were also prepared and digested. Lead was not detected in any of the laboratory blank wipe samples.

Reagent blanks were similarly digested and analyzed with the samples. Lead was not detected in any of the reagent blanks.

Certified Reference Materials (CRM) for soils (Institute for Reference Materials and Measurement and INTER 2000, France) were weighed and digested together with soil samples. Lead concentration in the CRM certificate was 64 mg/kg, while lead levels in the CRM digests ranged from 63.4 mg/kg – 64.0 mg/kg, equivalent to 98.98–99.97% recoveries of the certified concentration.

An inter-laboratory comparison of the lead levels in six randomly selected samples each of soil and house dust was conducted at the Kenya Industrial Research and Development Institute (KIRDI) and Kenya Plant Health Inspectorate Services (KEPHIS) Analytical Chemistry Laboratories in Nairobi using similar procedures. Lead was detected using atomic absorption spectrometry and graphite furnace atomic absorption spectrometry in KIRDI and KEPHIS, respectively. The correlation coefficient between the sets of soil and house dust lead levels was 0.94 and 0.99, respectively. The study results were therefore deemed to be reliable. Calibration curves are shown in the Supplemental Material.

### Estimation of Child Blood Lead Levels

From the measured floor dust lead loadings and soil lead concentrations, IEUBK version 1.1 Build 11 (Syracuse Research Corporation, North Syracuse, New York) was used to predict BLLs in children age ≤ 7 years.^18^ The IEUBK model has been widely used to predict blood lead concentrations in young children exposed to lead. The model mathematically and statistically links environmental lead exposure to blood lead concentrations for one child or a population of children between the ages 0–7 years. The model uses exposure, uptake, biokinetic, and probability distribution to estimate blood lead levels in children exposed to lead contaminated media. The geometric mean blood lead is predicted from available information about children's exposure to lead such as soil and dust data.^18^,^19^

The amount of lead in residential dust is quantified by lead loading (measured in micrograms per square meter (μg/m^2^) or micrograms per square foot (μg/ft^2^)), and lead concentration measured in micrograms per gram (μg/g or ppm). Dust lead concentration is calculated from lead loading and dust loading as follows: dust lead concentration = Lead loading/Dust loading.^24^ From this distribution, the model estimates the risk (probability) that a particular child or a population of children will have their blood lead concentrations exceed the Centers for Disease Control and Prevention reference values for lead in blood.^18^ For this study, the model assumed that soil and dust are the only major means by which children come in contact with lead. Therefore, all other lead pathway (maternal, air, water and diet) values were set to zero and only dust and soil values were inputted. The default soil ingestion value was set as 500 mg/day for the dust and dirt environments studied.

Currently, there are no reference levels for pediatric blood lead or house dust lead in Kenya. Therefore, 10 μg/dL and 5 μg/dL were used for comparison, while 40 μg/ft^2^ was used as a reference value for house dust lead loading on floors.^25–27^ United States Environmental Protection Agency (USEPA) guidance values of 400 mg/kg lead in soils in residential areas, schools and playgrounds, and 1,200 mg/kg lead in waste dumps and industrial soils were used.^28^

### Statistical Methods

Statistical analyses were performed using Minitab version 17.0 (Minitab Inc.) The Ryan-Joiner test was used to test the normal/log-normal distribution of the data for soil and floor dust lead values in the areas studied. All data were log-transformed. The geometric means and medians for soil and house dust were calculated. Comparison of lead concentrations in house dust in different sampling sites was done using one-way analysis of variance. Statistical significance was set at p<0.05, unless otherwise stated.

## Results

Lead was detected in 100% of floor dust samples. Each floor dust lead loading measurement from dwelling units and preparatory schools in the Dandora, Kariobangi and Mukuru slums exceeded the USEPA guidance value of 40 μg/ft^2^ (*Table 1*).^27^ In contrast, only 76.7% and 68.8%, respectively, of floor dust loadings from dwelling units and preparatory schools in the control area exceeded the USEPA guidance value.

**Table 1:** Lead Loadings (μg/ft^2^) from Floors in Dwelling Units and Preparatory Schools in the Study Areas

	**Dwelling Units**			**Preparatory Schools**		
*Study Area*	**N**	Geometric Mean^*^ ± SD	**Median**	**N**	**Geometric Mean ± SD**	**Median**
**Dandora**	30	7495.1 ±1.8^d^	7094.0	44	1749.0 ±1.57d	1617.3
**Kariobangi**	30	9358.1 ±2.31^e^	7390.9	56	2300.8 ±2.51d	2016.3
**Mukuru**	30	8892.8 ±1.74^e^	8839.7	55	1828.0 ±2.18d	2050.8
**Ruiru (Control)**	30	63.7 ±1.61^a^	75.3	48	46.3 ±2.65a	59.4
**F value**		451.5			257.8	
**P value**		<0.0001			<0.0001	

^*^Geometric mean lead loading values that do not share a superscript letter are significantly different. Abbreviations: SD, Standard deviation; N, Number of samples.

**Table 2:** Percentage Distribution of Floor Lead Loadings in the Study Areas

	**Sampling Sites**	**n**	**N**	**Percentage (%) of Floor Lead**		
				**Loading Measurements**		
*Study Area*				**≤10 μg/ft^2^**	**>10≤40 μg/ft^2^**	**>40 μg/ft^2^**
**Dandora**	Dwelling units	30	30	0.0	0.0	100.0
	Preparatory schools	10	44	0.0	0.0	100.0
**Kariobangi**	Dwelling units	30	30	0.0	0.00	100.0
	Preparatory schools	13	56	0.0	0.0	100.0
**Mukuru**	Dwelling units	30	30	0.0	0.0	100.0
	Preparatory schools	12	55	0.0	0.0	100.0
**TOTAL**		125	244	0.0	0.0	100.0
**Ruiru**	Dwelling units	30	30	0.0	20.0	80.0
**(Control)**	Preparatory schools	9	48	14.6	16.7	68.8
**TOTAL**		39	78	8.9	17.9	73.1

Abbreviations: N, Number of Samples; bn, Number of Sampling Sites; c40 μg/ft^2^, US EPA guidance value for lead loading in indoor floor dust.^27^

The lead loadings were highest in Kariobangi, followed by Mukuru slums, then Dandora. Indoor floor dust samples from Kariobangi recorded lead loadings as high as 58,194 μg/ft^2^, (about 6% lead) compared to the USEPA guidance value of 40 μg/ft^2^. The control area recorded lower mean lead loadings compared to the study areas.

Soils in the Dandora, Kariobangi and Mukuru slums were found to have high lead concentrations, as shown in Table 3. The recorded geometric mean soil lead concentrations were highest in the Mukuru slums, followed by Dandora, then Kariobangi. Waste dump and industrial soils in the Dandora, Kariobangi and Mukuru slums were found to have a high geometric mean lead concentration (2,630.5 mg/kg). The lead concentrations in the waste dump and industrial soils were elevated and ranged between 1,589.0 mg/kg and 7,108.0 mg/kg, over the USEPA guideline of 1,200 mg/kg for lead in waste dumps and industrial soils (*Table 3*).^28^ The study established that outdoor soils in preparatory schools, residences and playgrounds near informal ULAB recycling activities in Dandora, Kariobangi and Mukuru had a high geometric mean lead concentration of 437.1 mg/kg compared to the USEPA guidance value of 400 mg/kg for lead in soils in residential, playground and school areas, with a range of 214.0 mg/kg to 1,870.8 mg/kg. Lead concentrations in 57.5% (69 out 120) of the soil samples from residential, playgrounds and preparatory schools in Dandora, Kariobangi and Mukuru slums exceeded the 400 mg/kg standard. Control soil samples recorded low lead concentrations that were below the recommended values.

**Table 3:** Lead Concentrations in Composite Soil Samples by Site and Location

		**Dwelling Units**				
*Study Area*		**Waste dumps**	**Industrial sites**	**Residential areas**	**Playgrounds**	**Preparatory schools**
**Dandora**	Geometric Mean^*^ ± SD	1891.3 ± 1.0^a^	1933.9 ± 1.0^a^	472.2 ± 1.1^fg^	400.7 ± 1.0gh^i^	339.1 ± 1.3^hi^
	Median	1883.0	1922.0	463.6	398.7	331.9
	N	9	9	18	9	13
**Kariobangi**	Geometric Mean^*^ ± SD	1665.4 ± 1.0^b^	1670.7 ± 1.15^b^	391.9 ± 1.2gh^i^	357.1 ± 1.1^ghi^	440.5 ± 1.9^f^
	Median	1631.1	1654.0	395.4	349.3	380.0
	N	9	9	18	9	13
**Mukuru**	Geometric Mean^*^ ± SD	6922.9 ± 1.1^c^	4714.9 ± 1.1^d^	758.9 ± 1.1^e^	477.8 ± 1.1^fgh^	319.4 ± 1.3^i^
	Median	7097.0	4433.0	773.2	449.0	345.2
	N	9	9	18	9	13
**Ruiru (Control**	Geometric Mean^*^ ± SD	58.8 ± 1.0^j^	56.61 ± 1.1^j^	21.45 ± 1.3^j^	32.14 ± 1.6^j^	55.0 ± 1.4^j^
	Median	57.9	55.4	19.6	23.9	51.6
	N	9	9	18	9	13
**F value**		231.6	345.4	97.3	57.8	126.6
**p value**		<0.001	<0.001	0.004	0.0046	<0.001

^*^Geometric mean loading values that do not share a superscript letter are significantly different. Abbreviations: SD, Standard deviation; N, Number of samples.

Table 5 shows the predicted BLL in children ≤ 7 years old in the study areas. Children in Dandora, Kariobangi and Mukuru were predicted to have elevated mean BLLs that exceeded the Centers for Disease Control (CDC) reference value of 5 μg/dL for lead in blood.^26^ Children living near informal ULAB activities in the Mukuru slums were predicted to have the highest geometric mean blood lead level followed by those living in Dandora, Kariobangi and Ruiru, consecutively. Accordingly, children living in the control area (Ruiru) were predicted to have a low mean blood lead level below the CDC recommended value (*Table 5*).

**Table 4:** Percentage Distribution of Soil Lead Concentrations Across Study Areas

			**Percentage (%) of Soil Lead**			
			**Concentration Measurements (mg/kg)**			
*Study Area*	**Sampling Sites**	**N**	**≤40**	**>40≤100**	**≤1, 200**	**>1, 200**
**Dandora**	Waste dumps	9	0.0	0.0	0.0	100.0
	Industrial sites	9	0.0	0.0	0.0	100.0
**Kariobangi**	Waste dumps	9	0.0	0.0	0.0	100.0
	Industrial sites	9	0.0	0.0	0.0	100.0
**Mukuru**	Waste dumps	9	0.0	0.0	0.0	100.0
	Industrial sites	9	0.0	0.0	0.0	100.0
**Total**		54	0.0	0.0	0.0	100.0
**Ruiru (Control)**	Waste dumps	9	0.0	100.0	0.0	0.0
	Industrial sites	9	0.0	100.0	0.0	0.0
**Total**		18	0.0	100.0	0.0	0.0
			≤40	>40≤100	≤400	>400
**Dandora**	Residential areas	18	0.0	0.0	0.0	100.0
	Playgrounds	9	0.0	0.0	66.7	33.3
	Preparatory schools	13	0.0	0.0	84.6	15.4
**Kariobangi**	Residential areas	18	0.0	0.0	61.1	38.9
	Playgrounds	9	0.0	0.0	77.8	22.2
	Preparatory schools	13	0.0	0.0	61.5	38.5
**Mukuru**	Residential	18	0.0	0.0	0.0	100.0
	Playgrounds	9	0.0	0.0	0.0	100.0
	Preparatory schools	13	0.0	0.0	61.5	38.5
**Total**		120	0.0	0.0	42.5	57.5
**Ruiru (Control)**	Residential	18	100.0	0.0	0.0	0.0
	Playgrounds	9	66.7	33.3	0.0	0.0
	Preparatory schools	13	15.4	84.6	0.0	0.0
**Total**		40	65	35	0.0	0.0

Abbreviations: N, Number of Samples; 1,200 mg/kg, USEPA guidance value for lead concentration in waste dump and industrial soils; 400 mg/kg, USEPA guidance value for lead concentration in schools, playgrounds and residential soils.^28^

**Table 5:** Predicted Blood Lead Levels in Children = 7 Years Old Across Study Areas

	**Dandora**	**Kariobangi**	**Mukuru**	**Ruiru**
**Age (Years)**				
**0.5–1**	27.3	25.5	44.1	2.3
**1–2**	25.1	23.3	40.5	1.9
**2–3**	22.7	21.1	37.1	1.6
**3–4**	22.3	20.7	36.8	1.5
**4–5**	22.1	20.4	36.7	1.5
**5–6**	21.4	19.7	36.0	1.4
**6–7**	20.3	18.7	34.4	1.3
**GM**	22.4	20.8	37.0	1.6
**% exceedance (10 μg/dL cut off)**	95.7	94.0	99.7	0.0
**% exceedance (5 μg/dL cut off)**	99.9	99.9	99.9	0.8

IEUBK was used to predict the BLL; 10 μg/dL (Centers for Disease Control, 2002) and 5 μg/dL (Centers for Disease Control, 2012) were used as the cut off/reference for child BLL. Abbreviations: GM, Geometric mean blood lead level in children; ^*^Geometric standard deviation = 1.60. ^**^Child soil ingestion rate = 500 mg/day.^18^,^19^,^25^,^26^

## Discussion

**Figure 2:**
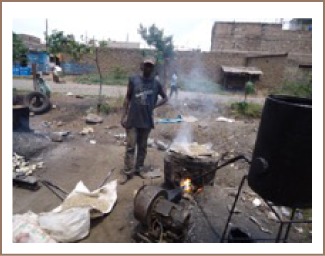
Informal ULAB recycling industrial site near a preparatory school in a residential area in Dandora (source: Ondayo, 2015).

**Figure 3:**
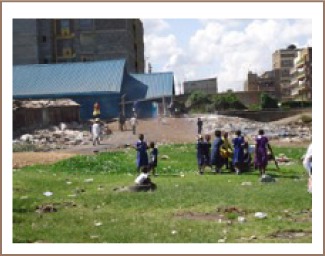
Informal ULAB recycling waste dumps near preparatory schools and children's playgrounds within a residential area in Kariobangi (source: Ondayo, 2015).

We found lead contamination exceeding USEPA reference values in outdoor soils and interior floor dust in children's environments located within two kilometers radius from informal ULAB recycling operations in the Dandora, Kariobangi and Mukuru slums. We hypothesize that this contamination is a result of the informal ULAB recycling activities that we observed being carried out, with no environmental or human exposure controls, in residential areas, near preparatory schools and near children's playgrounds in the study area (*Figure 2*); however, other sources of environmental lead contamination such as historical use of leaded petrol, spray painting, panel beating, metal cutting and welding as well as motor vehicle mechanics may have also contributed.^29^ Previous studies have shown that unregulated informal ULAB recycling activities are significant sources of indoor and outdoor lead contamination.^6^,^30^ Lead accounts for the majority of the weight of a lead-acid battery—about 70%. In addition, the crude methods that are used in informal ULAB recycling processes that include breaking of used batteries, removal of lead plates, crushing, screening, dry mixing, open burning and melting down generate large amounts of lead-containing dust, fumes and wastes.^1^,^4^,^8^,^31^ In our study communities, we observed ULAB recycling activities releasing white fumes which were blown by the prevailing winds (*Figure 2*) as well as ULAB recycling wastes dumped in the open near preparatory schools and children's playgrounds within residential areas (*Figure 3*). We also observed dust from the ULAB operations settling on surrounding soils and buildings. The lead contaminated soils and dust could become airborne when disturbed and blown by wind, causing widespread indoor and outdoor contamination.^32^,^33^ Family members can also take home lead-contaminated soil and dust from outside when they enter the house without removing their shoes and/or with contaminated work clothing.^32^

Additionally, we anecdotally observed that many floors in the dwelling units and preparatory schools in the Dandora, Kariobangi and Mukuru slums were dirty, with walls constructed using broken iron sheets with openings that may have allowed lead–containing dust to penetrate indoors. The walls and floor surfaces also had crevices into which lead containing dust could have embedded. We also anecdotally observed residents dry sweeping their dirty floors with children nearby, dust settled on toys, hands, pacifiers and similar objects, and children eating with dirty hands. Studies have shown that children's hand-to-mouth activity and pica behavior in lead contaminated environments is associated with elevated BLL.^9^,^21^ The USEPA estimates that the typical 1–6 year old American child ingests between 100–400 mg of soil and house dust every 24 hours, with the highest ingestion rate at the age of two years.^34^ In the dusty environments of our study communities, it may be reasonable to assume that children's soil and dust ingestion rates are even higher.

The mean lead loading in 73% of floor dust samples from dwelling units and preparatory schools in the control area (Ruiru) exceeded the USEPA 40 μg/ft^2^ guidance value for lead on floors.^27^ Only 27% of the control floor dust samples recorded lead loading values that were below the regulatory limit (*Table 1*). This could be attributed to the fact that lead particles are readily transported by air and wind, resulting in contamination of further away places.^35^

Studies have shown the correlation of soil and house dust lead to blood lead in support of pica and hand-to-mouth routes of lead ingestion in children.^9^,^11^,^36–38^ Based on this evidence, models such as the IEUBK are used to predict BLL from environmental lead levels. In validation studies, IEUBK's predicted BLLs were comparable with actual BLL.^19^,^37^ Using the IEUBK model, we estimated that 99.9% of children ≤ 7 years old living near informal ULAB recycling operations in Dandora, Kariobangi and Mukuru were likely to have lead poisoning, with predicted BLLs exceeding the US CDC 5 μg/dL reference value. The Mukuru slums resulted in predictive BLLs above 34 μg/dL for 99.9% of exposed children. Sources of uncertainty in predicting children's BLL from environmental lead measurements include the bioavailability of lead in a particular environmental matrix and other factors.^18^ Given the environmental lead contamination we measured, the BLLs we predicted were similar to the lead concentrations observed at Thiaroye Sur Mer, in Dakar, Senegal and Dong Mai Village in Vietnam, where similar operations are carried out.^6^,^30^,^32^

Currently, there is no safe level of exposure to lead.^3^,^39^ Even low BLLs from 2 μg/dL to 10 μg/dL have been reported to be associated with neurological damage in children.^26^ The costs associated with childhood exposure to lead can be quantified in the form of reduction in work performance and productivity as a result of IQ losses, increased health care costs and, behavioral and psychosocial problems, among others.^40–42^ These have negative impacts on individuals, populations, society and the entire economy of the country.^42^,^43^ While the present study clearly documented a serious lead exposure pathway in these communities, it did not establish that they had lead poisoning. This can only be done by blood lead testing. The high lead exposures need to be confirmed with BLL testing in future studies.

## Conclusions

We found lead contamination in outdoor soil and interior floor dust in the vicinities of informal ULAB recycling operations in the Dandora, Kariobangi and Mukuru slums of Nairobi, Kenya. This study provides baseline data on child lead exposure levels in the informal battery recycling sector in Kenya. We believe that the lead exposures measured in this study are representative of the lead exposures in similar and related setups in Kenya. In conclusion, the Dandora, Kariobangi and Mukuru slums face significant environmental health challenges with many children currently at risk of lead poisoning due to the unprecedented growth of informal ULAB recycling.

The soil and house dust results illustrate the need for coordinated action to decrease lead emissions from informal battery recycling in Nairobi slums and to remediate existing soils, particularly around battery workplaces and waste dumps. Child BLLs in the communities studied need to be tested in order to establish poisoning. Parents, guardians, teachers, and workers in institutions dealing with children should be encouraged to perform activities that have been shown to reduce children's lead exposures in lead-contaminated areas, including regularly wet-mopping floors and other housing unit components, regularly washing children's hands, pacifiers and toys, preventing children from playing in bare or contaminated soil, moving children's play areas, daycares and preparatory schools away from waste dumps and areas where informal ULAB recycling activities are carried out, encouraging children and other adults to remove shoes and lead-dust contaminated clothing when entering the house to prevent tracking lead into houses, properly feeding children and improving children's environments to reduce exposure to lead.^7^,^30^,^32^,^44^
