# Supplementary material for: Child Exposure to Lead in the Vicinities of Informal Used Lead-Acid Battery Recycling Operations in Nairobi Slums, Kenya
Source: J Health Pollut. 2016 Dec 22;6(12):15–25. doi: 10.5696/2156-9614-6.12.15 (PMC6221501; doi:10.5696/2156-9614-6.12.15)
Supplement: Supplementary file 1 [file Ondayo_SuppMaterial.doc]

Supplemental Material

Figure A shows the calibration curve generated by the inductively coupled plasma optical emission spectrometer (ICP-OES) during analysis of lead in the soil and house dust sample digests at Mines and Geological Department Laboratory. Regression

Equation: Absorbance = -0.03600 + (0.9935*Concentration); r2 = 0.9995.

Figure A: Calibration curve for the ICP-OES

Regression Equation: Absorbance = -0.03600 + (0.9935*Concentration); **r^2^** = 0.9995

Figure B shows the calibration curve generated by the atomic absorption spectrometer (AAS) during the analysis of lead in soil and house dust sample digests at KIRDI Laboratory during the inter-laboratory comparison. Regression equation; Absorbance = -0.002+ 0.002*Concentration; r2= 0.9924.

Figure B: Calibration Curve for the AAS

Regression equation; Absorbance = -0.002 + 0.002*Concentration; **r^2^**= 0.9924

Figure C shows the calibration curve generated by the Graphite Furnace Atomic Absorption Spectrometer (GFAAS) during the analysis of lead in soil and house dust samples at KEPHIS laboratories during the inter-laboratory comparison. Regression

equation: Absorbance = (0.009600*Concentration) + 0.01319; r2 =

0.9983.

Figure C: Calibration curve for the GFAAS at KEPHIS laboratory.

Regression equation: Absorbance = (0.009600*Concentration) + 0.01319; **r**^2^ = 0.9983
